# Spatial distribution and source identification for heavy metals in surface sediments of East Dongting Lake, China

**DOI:** 10.1038/s41598-022-12148-x

**Published:** 2022-05-13

**Authors:** Yi Yuan, Baolin Liu, Hao Liu

**Affiliations:** School of Marine Sciences, China University of Geosciences in Beijing, 29 Xueyuan Road, Haidian District, Beijing, 100083 China

**Keywords:** Environmental impact, Limnology

## Abstract

Dongting Lake is one of the most important inland freshwater lakes in China. To investigate the spatial distribution and seasonal variation characteristics of heavy metals (Cr, Co, Cu, Zn, Cd, and Pb) in the lake, 53 surface sediment samples were collected in the East Dongting Lake (ED Lake) in the wet and dry seasons. Results show Cr, Co, Cu, Zn, Cd, and Pb contents were 1.7 (1.9), 1.8 (2.0), 2.9 (3.0), 1.9 (1.9), 11.7 (13.1), and 2.0 (2.2)-fold of their geochemical soil background values of Hunan province (China) in the wet (dry) season. Spatial and seasonal heterogeneity could be found in the distribution of Cr, Co, Cu, Zn, and Pb in the surface sediments. The enrichment factor (EF) suggested that Cd has reached severe enrichment in the sediment. The result of the geo-accumulation index (${I}_{geo}$) indicated that Cr, Co, Cu, Zn, and Pb were at levels corresponding to low contamination, and moderately to highly polluted with Cd. Multivariate statistical analysis including pearson correlation analysis and principal component analysis was used for the identification of potential sources of the heavy metals in the sediments. The results showed that Cu, Zn, and Pb from the sediments of the East Dongting Lake in the wet and dry seasons were possibly anthropogenic sources, such as emissions from mining and smelting while Al, Fe, and Cr are attributed for natural sources. Cd enrichment in the sediments is influenced by both natural factors, and human activities in local areas.

## Introduction

Due to their toxic behavior, persistence, non-biodegradability, and bioaccumulation, heavy metals are considered as key environmental pollutants that need to be controlled^1,2^. Once absorbed by living organisms, heavy metals are difficult to degrade and can be transferred and accumulated along the food chain, ultimately posing a significant threat to human health^3,4^. The two main sources of heavy metals in the environment are geochemical reactions and anthropogenic activities. The main anthropogenic sources are industrial discharge, agricultural chemicals, and municipal sewage discharge. Residual heavy metals from human activities enter aquatic ecosystems through surface runoff, groundwater, and atmospheric deposition. The identification of anthropogenic or natural sources of heavy metals is important and can be used to assess the impact of human activities on ecosystems and to predict changes in the biological effects of heavy metals^5^. Studies have shown that only a small proportion of the heavy metals entering aquatic systems exist in the water column in a dissolved state, and most of them are adsorbed by the sediment in various ways (physical adsorption, chemical precipitation, etc.) and enriched in the sediment, resulting in a much higher concentration of heavy metals in the sediment than in the water column. There is the potential for heavy metals in the sediment to be re-released into the water column, which can lead to secondary contamination of the water column when hydrodynamic conditions or environmental factors (pH, redox potential, etc.) change^6–8^. Therefore, sediment can be also considered as a potential source of heavy metals in the aquatic environment^9,10^. The seasonal variation of climate characteristics like temperature, rainfall, and humidity activities and production change significantly, which contributes to seasonally variable distribution characteristics and sources of heavy metals^11,12^. Hence, monitoring programs covering different seasons depict temporal variations in aquatic environment and may offer representative data for assessing the environmental status of the lake system^13^.

Dongting Lake is one of the most important inland freshwater lakes in China. Located in the eastern part of the Dongting Lake, East Dongting Lake (ED Lake) receives a constant flow of water and material input from the Yangtze River through the mouth of Ouchi and out through Chenglingji. The Dongting Lake is surrounded by a large number of industrial enterprises including metallurgical and chemical industries, as well as large areas of agricultural land^14^, and a large amount of industrial and agricultural wastewater, as well as domestic sewage, is discharged into the Dongting Lake every year^15,16^. Anthropogenic activities have greatly influenced the content and distribution of heavy metals in the lake. Many studies have been done on the investigation, monitoring, and assessment of the pollution status of Dongting Lake^17–19^. Annual precipitation in the Dongting Lake basin is 1100–1400 mm, which descends from the outer hills to the inner plains. The precipitation of mostly heavy rain and rainstorm from April to July provides over 50% of the total annual precipitation. April to August is the wet season in the area, with the maximum water level occurring from July to August. By contrast, November to March is the dry season^20^. The grain size and sedimentation rate of particles carried by the incoming water, and flow rate of the water body in the Dongting Lake during the wet season are different from those during the dry season, which can affect the content, distribution, and geochemical behavior of heavy metal elements in the Dongting Lake in different seasons^5,21^. However, most studies of heavy metals in Dongting Lake focused only on one season. Li et al. investigated heavy metals in the surface sediments of Dongting Lake in January and studied their spatial risk assessment and source apportionment^22,23^. Similarly, Peng et al. assessed the spatial distribution and ecological risk of heavy metals in West Dongting Lake in the dry season^24^. Few studies have compared the heavy metal content of Dongting Lake in the different seasons. In this study, ecological risk assessment and source apportionment of the sediments of the East Dongting Lake were carried out, and the spatial distribution of heavy metal levels in different seasons was compared.

The objectives of this study are (1) to investigate the seasonal variations (wet and dry seasons) of heavy metals content in the sediment of the ED Lake, (2) to conduct a source identification of heavy metals in the sediment of the ED Lake by using multivariate statistical analysis.

## Materials and methods

### Sample collection and analysis

**Figure 1:**
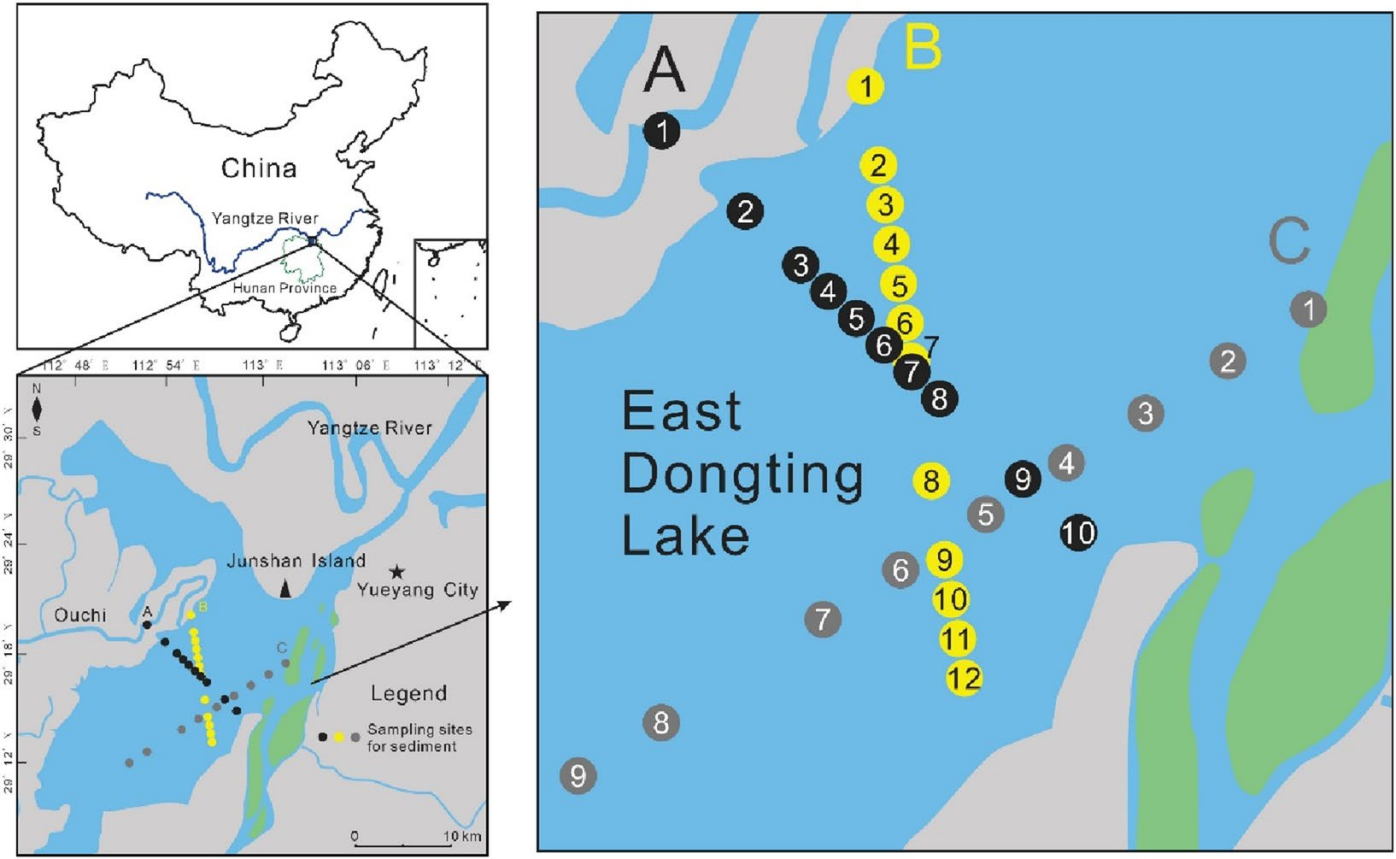
The geographic location of study area and sampling sites. Black, yellow, and gray solid dots represent sampling sites for sediment in this study.

Three cruises (A, B, and C) were designed for sampling to investigate the spatial distribution characteristics of heavy metals and for seasonal comparisons. Sampling was carried out along cruises A, B, and C in August 2012 (wet season) and along cruises A and B in January 2013 (dry season) (Fig. 1). A total of 53 surface sediment samples (31 samples in the wet season and 22 samples in the dry season) were collected using a homemade mud collector and placed in clean plastic bags. Samples collected in the wet and dry seasons were abbreviated as ED-W (including samples from cruises A, B, and C) and ED-D (including samples from A and B), respectively. The sampling sites were kept at the same latitude and longitude coordinates during the dry season as during the wet season.

The sediment samples were naturally dried and ground through a 200 mesh sieve. All samples were stored at 4 °C and analyzed at the Beijing Research Institute of Uranium Geology (BRIUG). Cr, Co, Cu, Zn, Cd, and Pb in the sediments were determined by inductively coupled plasma mass spectrometry (ICP-MS, ELEMENT XR, Thermo, USA). Al and Fe were determined by X-ray fluorescence spectrometer (XRF, AB-104 L, PW2404, Ltd., the Netherlands).

### QA/QC

Sample duplicates, method blanks, and standard reference materials GBW07309 (GSD-9) and GBW07312 (GSD-12) were used for quality assurance and quality control. A duplicate experiment was done for every four samples and the pass rate of duplicate samples was 100%. The recoveries of the two standard reference materials were 96–106% and 103–106% respectively.

### Enrichment factor and geo-accumulation

The enrichment factor (EF) was calculated to estimate the enrichment and possible anthropogenic impact of heavy metals on sediments using the following Eq. ^25^:

$$EF=\frac{{(\frac{{C}_{i}}{{C}_{Al}})}_{Sediment}}{{(\frac{{C}_{i}}{{C}_{Al}})}_{Background}}$$

where ${(\frac{{C}_{i}}{{C}_{Al}})}_{Sediment}$ is the ratio of the concentration of a particular metal i (C_i_) to the Al concentration (C_Al_) in the sediment sample; and ${(\frac{{C}_{i}}{{C}_{Al}})}_{Background}$ is the ratio of the background concentration of a particular metal i (C_i_) to the reference background concentration of Al (C_Al_). Either Al or Fe has been used as a conservative element for EF calculations in many studies to distinguish the source of heavy metals^26,27^. In this study, Al was selected as a reference metal. The value of enrichment factor < 1 indicates no enrichment; 1 to 3 is minor; 3 to 5 is moderate; 5 to 10 is moderately severe; 10 to 25 is severe; 25 to 50 is very severe, and > 50 is extremely severe enrichment^28^.

The geo-accumulation (${I}_{geo}$) method is a quantitative index for the study of heavy metal pollution in sediments^29^. ${I}_{geo}$ was calculated as:

$${I}_{geo}={log}_{2}[{C}_{i}/(k\times {B}_{i})]$$

where ${C}_{i}$ is the concentration of heavy metal i in the samples, mg/kg; ${B}_{i}$ is the soil background value of heavy metal i in Hunan province, China^30^. A factor of 1.5 is used to amend the possible variation in the background value due to lithogenic effects. The classification of ${I}_{geo}$ was as follows: unpolluted (${I}_{geo}\le 0$), lowly polluted (${0<I}_{geo}\le 1$), moderately polluted (${1<I}_{geo}\le 2$), moderately to highly polluted (${2<I}_{geo}\le 3$), highly polluted (${3<I}_{geo}\le 4$), highly to extremely polluted (${4<I}_{geo}\le 5$), and extremely polluted (${I}_{geo}>5$).

### Statistical analysis

Pearson correlation analysis and principal component analysis were performed by SPSS 22.0 (IBM SPSS Inc.). Pearson correlation analysis was subjected to a two-tailed significance test. Kaiser–Meyer–Olkin (KMO) and Bartlett's sphericity test were used to assess the validity of the principal component analysis^31^. The varimax method was used for rotation. Extraction of principal components based on eigenvalues (> 1) and cumulative variance (> 80%) in principal component analysis.

## Results and discussion

### Heavy metals contents

As can be seen from Table 1, in the ED-W, the concentrations of Zn varied the most between sites, ranging from 133.0 to 214.0 μg/g, while Co and Cd varied the least, with SD values of 1.34 and 0.47, respectively. In the ED-D, Zn and Cu had larger SD values than the other heavy metals (23.37 and 20.18, respectively), while Cd ranged from 0.48 to 2.94 μg/g with an SD value of 0.51. The contents of all six heavy metals in the sediments were higher than their background values to varying degrees. In the ED-W, Cr, Co, Cu, Zn, Cd, and Pb concentrations in the sediments were 1.7, 1.8, 2.9, 1.9, 11.7, and 2.0 times higher than those in the background values, respectively, while in the ED-D, Cr, Co, Cu, Zn, Cd, and Pb concentrations in the sediments were 1.9, 2.0, 3.0, 1.9, 13.1, and 2.2 times higher than those in the background values, respectively (Table 1).

**Table 1:** Concentrations (ω/(μg/g)) of heavy metals in sediment from the East Dongting Lake in the wet and dry seasons and comparison with other lakes.

Name of the Lakes		Heavy metals concentrations (mg/kg)					
		Cr	Co	Cu	Zn	Cd	Pb
ED-W^a^ (n = 31)	Min	93.5	19.9	54.6	133.0	0.3	32.9
	Max	122.0	25.5	95.3	214.0	2.1	78.9
	Mean	109.4	22.82	72.62	171.42	0.92	55.54
	SD	6.10	1.34	11.24	22.92	0.47	14.86
ED-D^b^ (n = 22)	Min	91.8	21.1	50.0	117.0	0.48	34.5
	Max	134.0	30.0	112.0	201.0	2.94	84.1
	Mean	121.63	25.82	77.20	164.77	1.03	61.13
	SD	10.18	2.11	20.18	23.37	0.51	14.28
Reference values^c^		64.9	12.9	25.4	88.6	0.0787	27.3
Dongting Lake, China^22^		88.29		47.48	185.75	4.65	60.99
Honghu Lake, China^32^		25		78	145	0.14	20.66
Yilong Lake, China^33^		86.73		31.4	86.82	0.76	53.19
Veeranam Lake, India^34^		88.2		94.12	180.08	0.81	30.06
Hussain Sagar Lake, India^35^		90		90.108	273.14	19.89	79.885
Zariwar Lake, Iran^36^		74.41	9.28	16.97	34.23	0.25	
Lake Van, Turkey^37^		46		20	22	ND^d^	5
Lake Karla, Greece^38^		320	38.6	41.7	18.3		29.1
East Dongting Lake, China^32^		33.06		46.35	154.63	2.74	35.15

^a^East Dongting Lake in the wet season.

^b^East Dongting Lake in the dry season.

^c^Soil background values in Hunan province, China^30^.

^d^Not detected.

A comparison of the heavy metal contents of the sediments in the East Dongting Lake with those in some other lakes is shown in Table 1. High concentrations of Cr, Cu, Zn, Cd, and Pb can be found in some lakes, possibly closely related to local anthropogenic activities. Another study on the East Dongting Lake showed a significant decrease of concentrations in Cr, Cu, Zn, and Pb compared to this study, except for a significant increase of concentrations in Cd from 0.92 mg/kg and 1.03 to 2.74 mg/kg in the ED-W and ED-D.

### Spatial distribution of heavy metals in different seasons

**Figure 2:**
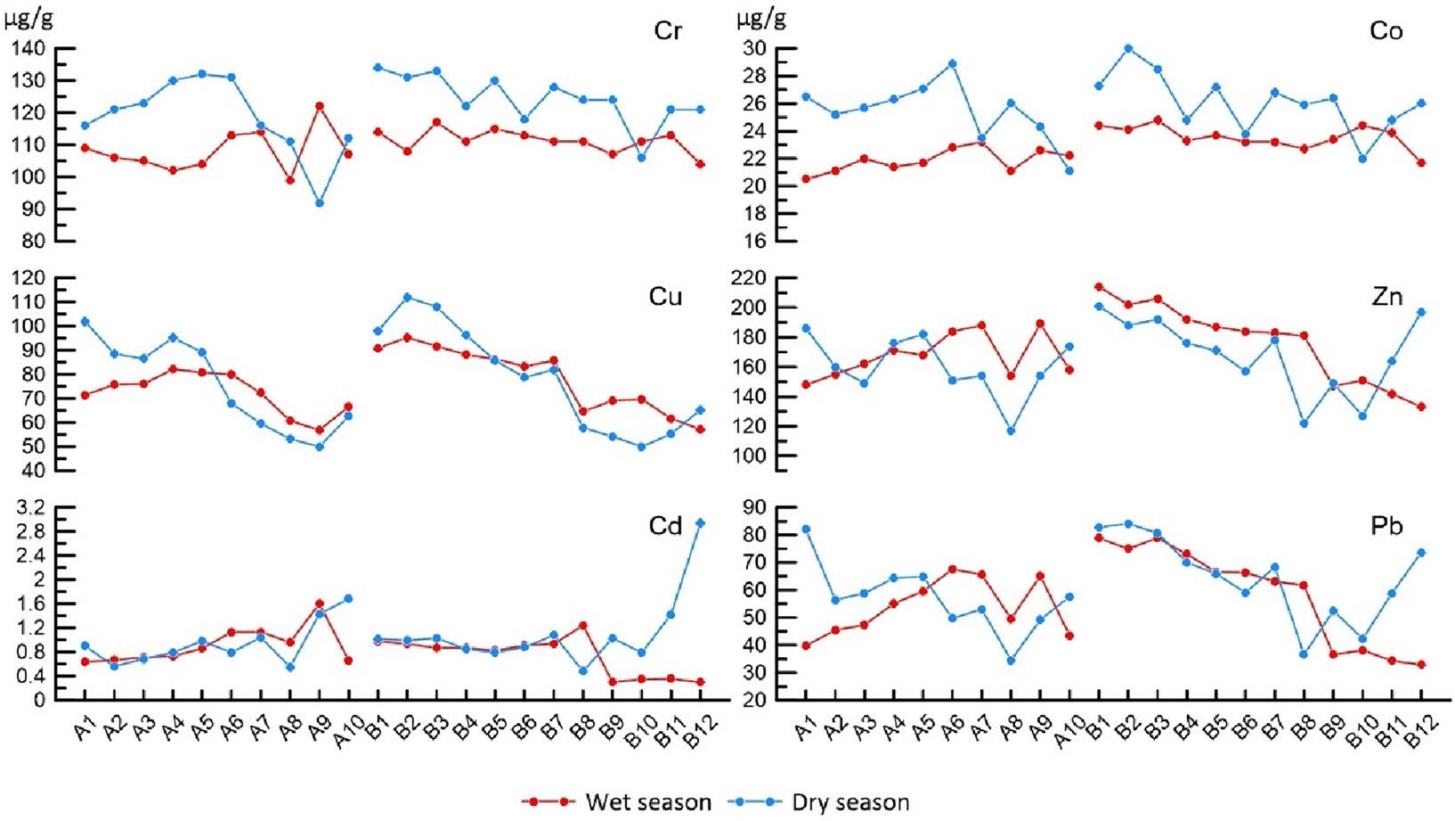
Heavy metals contents in the sediments from cruises A and B in the ED Lake (μg/g). A1 to A10 belongs to cruise A, while B1 to B12 belongs to cruise B. Red solid dots represent samples collected in the wet season, and blue solid dots represent samples collected in the dry season.

According to Fig. 2, The peak concentrations of heavy metals Cr, Co, Cu, Zn, and Pb in the dry and wet seasons were found at sites B1, B2, and B3 (except Cr in the wet season), while the valley values were found at sites A10, A9, A8, and B12 (except Co in the wet season). In cruise B (from B1 to B12), a clear trend of variation of Cu, Zn, and Pb contents with spatial distribution can be observed. In cruise A, this variation can only be clearly seen in Cu. As can be seen in Fig. 1, the sites B1, B2, and B3 are close to the left shore of the lake and are located near the outlet of Ouchi, from which the water flow from the Yangtze River is discharged into the East Dongting Lake. Therefore, this spatial distribution of heavy metal content was most likely to be affected by hydrodynamic conditions^39^. During the flow of water from the outlet of Ouchi to the center of the lake, the velocity of the water gradually decreases, and the sediment carried by the water is gradually deposited on the bottom of the lake, together with the dilution effect of the lake water, thus the heavy metal content decreases with the increase of the offshore distance. Some elements, such as Pb and Zn, showed an increasing trend of content from A1 to A10 in the wet season samples. It might be attributed to the fact that aquatic plants are more abundant near the shore due to the shallow water depth. Some plants have an enrichment behavior towards heavy metals in the sediment and this may have an impact on the heavy metal content in the sediment. Differences in the growth of aquatic plants at different sites were observed during the field survey. Additionally, as for the cruise C, however, unfortunately, no samples were collected in the dry season. Only the concentration of the heavy metals in the wet season was summarized here briefly. The variation of heavy metal content with spatial location can be observed from C1 to C9. There is an increasing trend of Cd, Pb, and Zn content from C3 to C9. The heavy metal content in the cruise C samples is close to that of the wet season samples from cruises A and B. However, the spatial distribution pattern of heavy metal contents of the cruise C samples is somewhat different from that of the wet season samples from cruises A and B. The distribution of Co, Zn, and Pb contents in cruise C tended to be similar to that of the wet season samples from cruise A rather than the wet season samples from cruise B.

The mean concentrations of Cr, Co, Cu, Cd, and Pb were higher in the dry season than in the wet season probably due to the dilution by rainwater (Table 1), which influences concentration and heavy metal mobility. Furthermore, the mobility of heavy metals depends not only on the total concentrations in the sediment, but also on the soil or sediment properties themselves, metal properties, and environmental factors^40^. In addition, aquatic plants flourish during the wet season and aquatic plants can have an impact on the heavy metal content of the sediment. Some aquatic plants can take up heavy metals from the sediment, which can lead to a reduction in the heavy metal content of the sediment^41^. On the contrary, Zn has lower mean values in the dry season than in the wet season, which can be attributed to the large amount of rain in the wet season, precipitation causes more pollutants on the land to flow into the river and settle in the sediment^39^. In addition, higher SD values of Cr, Co, and Cu are observed in the wet season than in dry seasons, indicating the greater variations in the contents of Cr, Co, and Cu.

### Assessment of sediment risk

Based on EF values (Table 2), the majority of elements (Cr, Co, Cu, Zn, and Pb) showed no or minor enrichment at all sites, indicating lighter anthropogenic pollution of the sediment. Only Cd show higher EF values. The maximal EF value for Cd in ED-W and ED-D are 14.15 and 18.87, respectively; according to the EF scale, they belong to severe enriched sediments. Hence, the significant enrichment of Cd may pose a more serious potential ecological risk to the surrounding environment.

**Table 2:** The EF values of six heavy metals in sediments from the East Dongting Lake in the wet and dry seasons.

EF		Cr	Co	Cu	Zn	Cd	Pb
ED-W^a^ (n = 31)	Min	0.77	0.71	0.91	0.78	1.97	0.63
	Max	1.05	1.08	2.17	1.32	14.15	1.62
	Mean	0.89	0.93	1.51	1.02	6.07	1.07
ED-D^b^ (n = 22)	Min	0.65	0.87	0.91	0.72	3.4	0.69
	Max	1.15	1.32	2.5	1.2	18.87	1.75
	Mean	1	1.07	1.64	0.99	6.92	1.2

^a^East Dongting Lake in the wet season.

^b^East Dongting Lake in the dry season.

As can be seen from Table 3, all the average ${I}_{geo}$ values of Cr, Co, Cu, Zn, Cd, and Pb were above zero, indicating that sediment of the wet and dry seasons was in polluted status. From the classification criteria, the sediments could be categorized as lowly polluted with Cr, Co, Cu, Zn, and Pb (0 < mean values < 1), and moderately to highly and highly polluted with Cd in the wet and dry seasons (mean values = 2.77 and 3.01), respectively.

**Table 3:** The ${I}_{geo}$ value and series of six heavy metals in sediments from the East Dongting Lake in the wet and dry seasons.

${I}_{geo}$		Cr	Co	Cu	Zn	Cd	Pb
ED-W^a^ (n = 31)	Min	−0.06	0.04	0.52	0.00	1.34	− 0.32
	Max	0.33	0.40	1.32	0.69	4.14	0.95
	Mean	0.17	0.24	0.91	0.35	2.77	0.39
	PG^c^	Lowly polluted	Lowly polluted	Lowly polluted	Lowly polluted	Moderately to highly polluted	Lowly polluted
ED-D^b^ (n = 22)	Min	−0.08	0.12	0.39	−0.18	2.02	− 0.25
	Max	0.46	0.63	1.56	0.60	4.64	1.04
	Mean	0.32	0.41	0.97	0.30	3.01	0.54
	PG^c^	Lowly polluted	Lowly polluted	Lowly polluted	Lowly polluted	Highly polluted	Lowly polluted

^a^East Dongting Lake in the wet season.

^b^East Dongting Lake in the dry season.

^c^Pollution grade.

### Multivariate statistical analysis

#### Pearson’s correlation

The values of the pearson correlation coefficient matrix are listed in Table 4. The results show that Al, Fe, and Cr were significantly positively correlated with each other (*p* < 0.01), and Cu, Zn, and Pb were significantly positively correlated with each other (*p* < 0.01) in the ED-W. In addition, Co was significantly correlated with Fe and Cr (*p* < 0.01). In the ED-D, Cr was significantly positively correlated with Cu, and Pb (*p* < 0.01), and Co was significantly positively correlated with Cu and Pb (*p* < 0.01). Cu, Zn, and Pb were significantly positively correlated with each other (*p* < 0.01). In addition, Fe was significantly correlated with Al (*p* < 0.01). Al and Fe are mainly found in fine-grained sediments and clastic minerals, and the strong correlation of heavy metals with Al and Fe suggests that these heavy metals are less influenced by anthropogenic activities and that they may be of the same origin as Al and Fe^42^.

**Table 4:** Pearson correlation coefficients between concentrations of eight elements in sediments from the East Dongting Lake in the wet and dry seasons.

		Al	Fe	Cr	Co	Cu	Zn	Cd	Pb
ED-W^a^ (n = 31)	Al	1							
	Fe	0.693**	1						
	Cr	0.496**	0.481**	1					
	Co	0.265	0.697**	0.666**	1				
	Cu	−0.371*	− 0.047	0.290	0.397*	1			
	Zn	0.215	0.258	0.640**	0.414*	0.621**	1		
	Cd	0.379*	0.087	0.374*	− 0.076	−0.043	0.661**	1	
	Pb	0.177	0.180	0.537**	0.290	0.616**	0.977**	0.677**	1
ED-D^b^ (n = 22)	Al	1							
	Fe	0.584**	1						
	Cr	−0.230	0.194	1					
	Co	− 0.022	0.556**	0.709**	1				
	Cu	−0.369*	0.019	0.636**	0.576**	1			
	Zn	0.173	0.100	0.477*	0.369*	0.700**	1		
	Cd	0.482*	−0.006	− 0.163	−0.157	−0.205	0.475*	1	
	Pb	−0.018	0.143	0.515**	0.469**	0.825**	0.943**	0.295	1

^a^East Dongting Lake in the wet season.

^b^East Dongting Lake in the dry season.

*Correlation is significant at the 0.05 level (2-tailed).

**Correlation is significant at the 0.01 level (2-tailed).

#### Factor analysis and source identification of heavy metals

**Figure 3:**
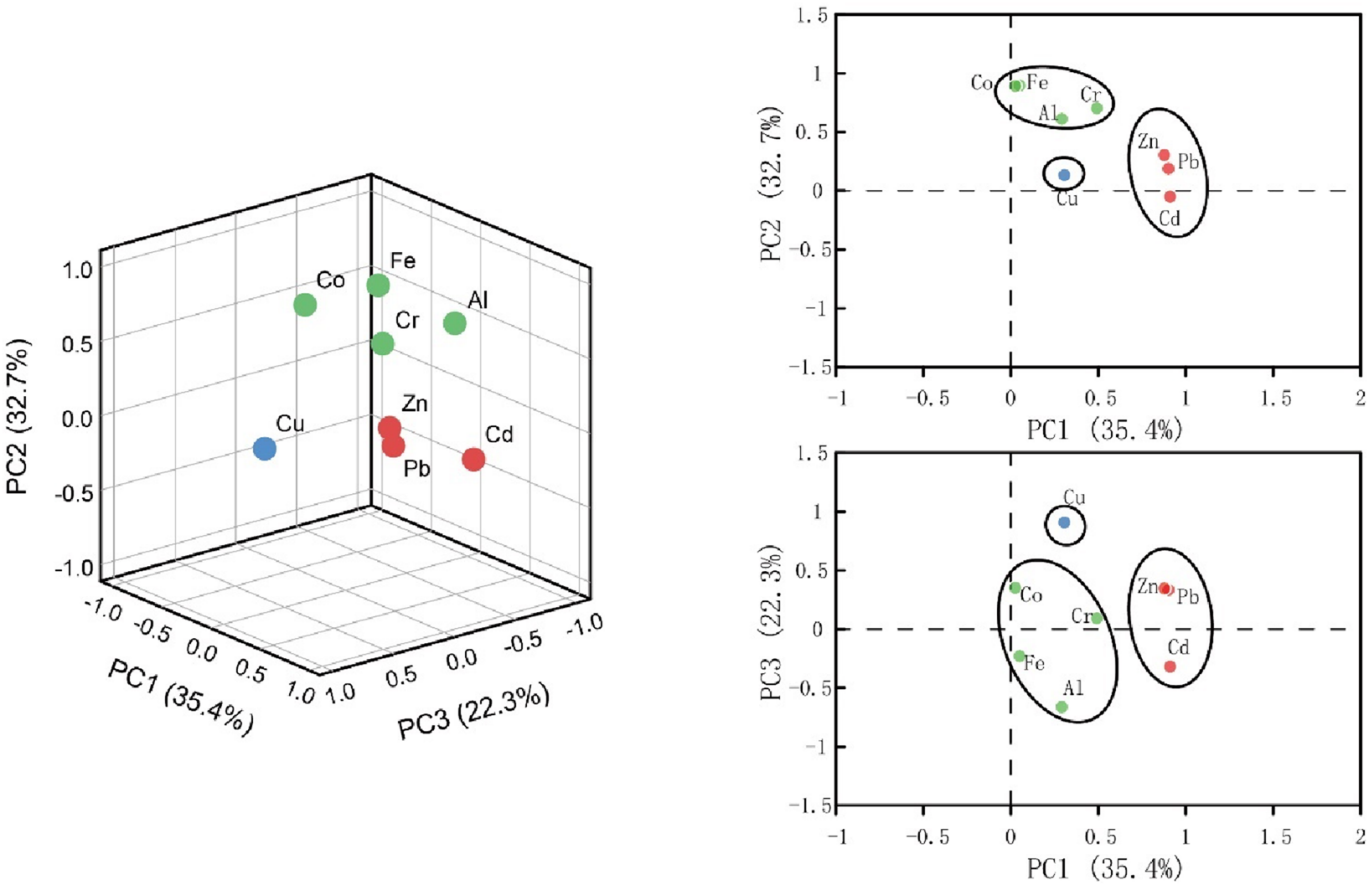
PCA results of the elements in the ED-W. Left: 3D loading plot of the elements; right: 2D loading plots on PC1–PC2 and PC1–PC3.

**Figure 4:**
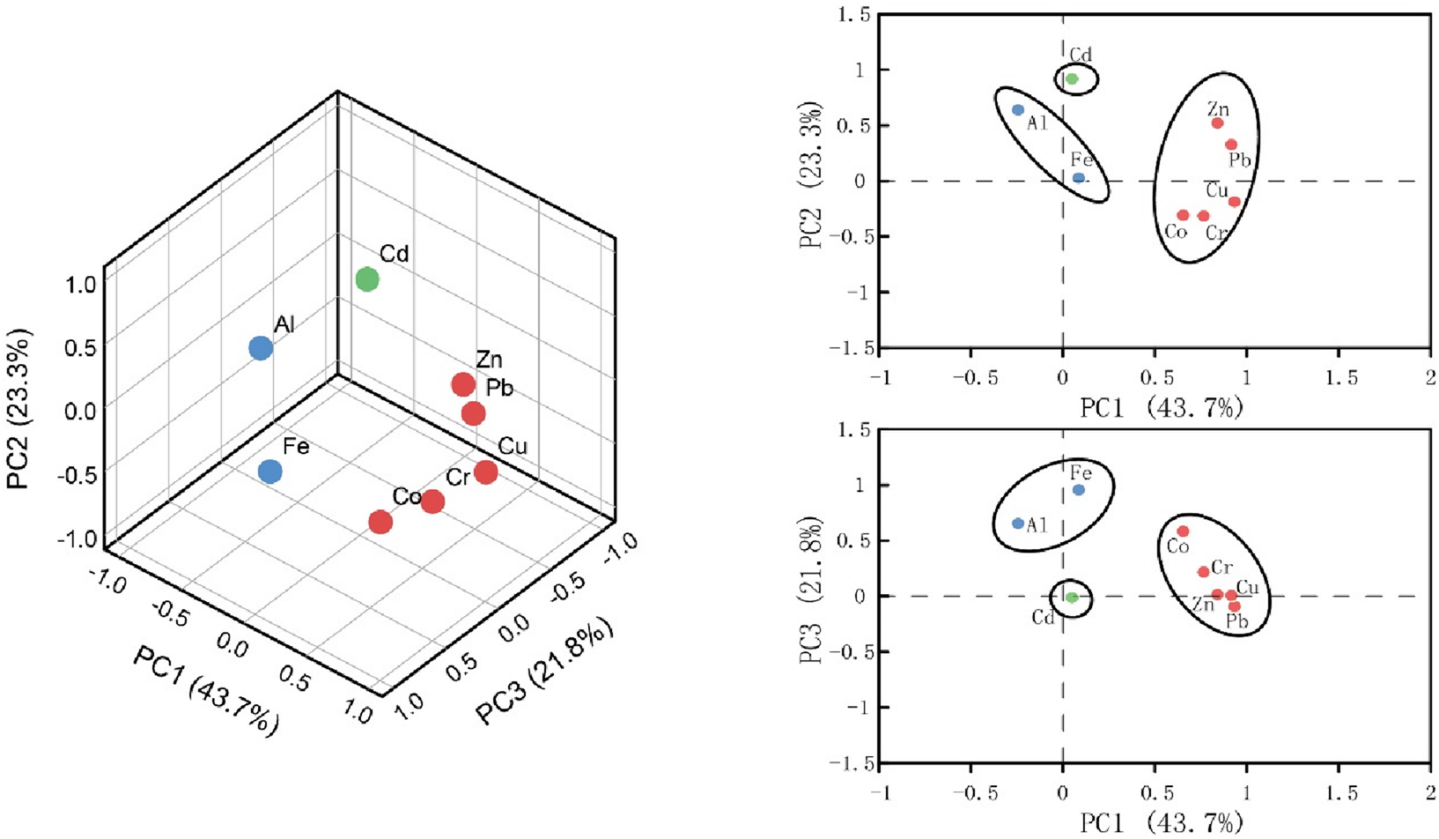
PCA results of the elements in the ED-D. Left: 3D loading plot of the elements; right: 2D loading plots on PC1–PC2 and PC1–PC3.

Principal component analysis (PCA) was performed on Al, Fe, Cr, Co, Cu, Zn, Cd, and Pb. Data for the eight elements can be reflected by three principal components. In the ED-W, the three principal components (PCs) accounted for 90.37% of the variability of all the studied variables. The result of PCA was represented in Fig. 3. Factors with high loadings (> 0.8) in PC1 are Cd, Pb, and Zn, and factors with high loadings (> 0.6) in PC2 are Fe, Co, Cr, and Al, and factor with high loading (> 0.9) in PC3 is Cu. Figure 4 shows the loading plots of the PCs in the ED-D, which have captured most of the variation from the data (88.744%). The loading plots show that the elements were discriminated into three clusters. Factors with higher loadings (> 0.6) in PC1 are Cr, Cu, Zn, Pb, and Co, and factor with higher loading (> 0.7) in PC2 is Cd, and factors with higher loadings (> 0.6) in PC3 are Al and Fe. Hunan province is known as the home of non-ferrous metals, with reserves of Pb and Zn among the highest in China, and considerable reserves of Cu. Activities such as mining and smelting can result in the accumulation of heavy metals in the surrounding environment^43^, and the significant positive correlation of Cd with Zn and Pb may be related to non-ferrous minerals such as Pb–Zn ores in the Yangtze River basin^44^. Some studies have shown the presence of Cd in pesticides and fertilizers, so Cd may be associated with agricultural production^45^. Municipal waste and domestic sewage are anthropogenic sources of Cu^46^. The significant positive correlation of Co with Cu and Pb in the ED-D suggests that Co may be of the same source as Cu and Pb. In addition, Cr released from the burning of coal in industrial production could enter the soil and sediment by way of deposition and the use of fertilizers and pesticides is also an essential way in which Cr and Cu enter the environment^47,48^. The results of a geochemical investigation on the Yangtze River Basin since 1999 also show that there are geochemical anomalies of Cd and other heavy metals in the alluvial deposits along the river banks, possibly mainly due to the weathering and denudation of Cd-rich rock layers^49,50^. For example, black shale enriched in Cd is widely distributed in the Yangtze River basin^51,52^. This also explains the moderate positive correlation of Cd with Al and Fe. In addition, industrial and agricultural activities also contribute to the enrichment of Cd^53^. In the ED-W, due to the significant positive correlation of Fe with Al and Co, and the strong positive correlation of Cr with Co (r = 0.625), Cr and Co may be from the same source as Al and Fe, i.e. natural erosion. Therefore, in the ED-W, PC1 may be interpreted as anthropogenic sources (like Cu, Zn, and Pb), including industrial activities such as mining and smelting and the emission and use of chemicals such as fertilizers and pesticides, PC2 may be interpreted as natural source (like Fe, Co, Cr, and Al), and PC3 may be interpreted as domestic sewage, like Cu. In the ED-D, PC1 may be interpreted as mining and agricultural production sources (like Zn, Pb, Cu, Cr, and Co). PC2 may be interpreted as natural weathering and agricultural activities, like Cd. PC3 may be interpreted as a natural source, like Al and Fe.

## Conclusion

This study analyzed the spatial distribution and identified the sources of six heavy metals (Cr, Co, Cu, Zn, Cd, and Pb) in the surface sediment of ED Lake. All the heavy metals (Cr, Co, Cu, Zn, Cd, and Pb) studied show higher contents than the corresponding soil background values of Hunan province (China) in varying degrees. The obvious spatial heterogeneity of the distribution of Cu, Zn, and Pb contents in ED-W and ED-D may result from the hydrodynamic conditions of water flow and biological effects. Moreover, seasonal variation had significant effects on the concentrations of heavy metals in the surface sediment, and differences in precipitation between the wet and dry seasons, as well as the growth of aquatic plants, may be responsible. The metal enrichment factor (EF) and geo-accumulation index (${I}_{geo}$) indicated a low contamination level of Cr, Co, Cu, Zn, and Pb in the study area and a moderate to severe enrichment of Cd in some sediment samples. The results of pearson’s correlation analysis and principal component analysis indicate that the heavy metals in ED-W and ED-D may be from anthropogenic sources (like Cu, Zn, Co, and Pb), and natural sources (like Al, Fe, Co, and Cr), while Cd may derive from natural weathering and some human activities.

## Data Availability

The datasets generated during and/or analysed during the current study are available from the corresponding author on reasonable request.
